# Combining Laboratory and Imaging Evaluation for Cardiovascular Risk Stratification in Systemic Lupus Erythematosus

**DOI:** 10.3390/jcm14145085

**Published:** 2025-07-17

**Authors:** Chrysanthi Staveri, Vassiliki Vartela, Sophie I. Mavrogeni, Stamatis-Nick C. Liossis

**Affiliations:** 1 Division of Rheumatology, Department of Internal Medicine, Patras University Hospital, GR 26504 Patras, Greece; snliossis@med.upatras.gr; 2 Onassis Cardiac Surgery Center, GR 17674 Athens, Greece; vasvartela@yahoo.gr; 3 University Research Institute of Maternal and Child Health, National and Kapodistrian University of Athens School of Medicine, Aghia Sophia Children’s Hospital, GR 11527 Athens, Greece; sophie.mavrogeni@gmail.com; 4 Division of Rheumatology, Department of Internal Medicine, University of Patras School of Medicine, GR 26500 Patras, Greece

**Keywords:** cardiovascular risk, systemic lupus erythematosus, genetic factors, auto-antibodies, magnetic resonance imaging

## Abstract

Systemic lupus erythematosus (SLE) is a multisystem auto-immune disease that may affect any organ/system, including the cardiovascular system. Several studies have shown that SLE is associated with an increased risk of cardiovascular disease (CVD), even though most of the patients who have lupus are young women. In this review, we present that apart from the traditional risk factors, there are more appropriate SLE-related indices such as imaging parameters, auto-antibodies, disease manifestations, medications, and genetic factors that might represent useful tools to create an algorithm for early identification of SLE patients at increased risk of CVD. Early recognition and appropriate treatment of patients at increased CVD risk might reduce morbidity/mortality and improve the quality of life of patients with SLE.

## 1. Introduction

The heart is one of the most frequently affected organs in SLE. Pericardium, myocardium, coronary arteries, valves, and the conduction system, all of them in parallel or separately, can be involved [1].

The clinical presentation of cardiovascular disease (CVD) varies from mild, moderate to severe. In this narrative review, we present the association between SLE and various forms of CVD, including coronary artery disease (CAD), myo-pericarditis, pericarditis, valvular heart disease, and vasculitis [2]. The main manifestations of cardiovascular involvement in SLE include:

### 1.1. Libman–Sacks Endocarditis

Libman–Sacks endocarditis, also known as nonbacterial thrombotic endocarditis (NBTE) or marantic endocarditis, includes a broad spectrum of pathologies ranging from very small particles seen only microscopically to large sterile vegetations on previously normal heart valves. It involves most often the aortic and mitral valves. Libman–Sacks endocarditis initially appears as an endothelial injury, due to a hypercoagulation state, such as solid tumor, SLE, and antiphospholipid antibody syndrome (APS). There are no guidelines for the management of these manifestations, and the therapeutic approach remains empiric [3].

### 1.2. Pericarditis

The evaluation of 1000 SLE assessed that 16% of them had serositis, including pleuritis and/or pericarditis [4]. The incidence of pericarditis ranges in the literature between 11 and 54% [5,6,7]. The reported incidence of clinically symptomatic pericarditis was 25%. However, asymptomatic pericarditis has been detected by echocardiography in more than 50% of SLE patients [8].

Hemolytic anemia, proteinuria, lymphadenopathy, and anti-Smith (anti-Sm) antibodies were associated with pericarditis, whereas pulmonary fibrosis and gastrointestinal infarction were associated only with pleurisy. Fever, Raynaud’s phenomenon, and anti-double-stranded DNA (anti-dsDNA) antibodies were associated with both pericarditis and pleurisy [5]. Male gender and younger age were associated with elevated risk of serositis and pericarditis [9,10]. In addition to the association with anti-Sm antibodies [5,10,11,12,13], other studies showed a link with anti-Jo1 [11], anti-dsDNA [7,13], and anti-ribonucleoprotein (anti-RNP) [10,13] to serositis/pericarditis. A study of pediatric SLE showed a predisposition to serositis with antibodies to anti-chromatin, anti-ribosomal P, and anti-La, in addition to anti-RNP, anti-Sm, anti-dsDNA, and anti-Ro [13].

Acute pericarditis is characterized by serofibrinous or fibrinous changes to the pericardium, which becomes fibrotic in chronic pericarditis [14]. Vascular proliferation is demonstrated on histology in patients with acute pericarditis [15].

The clinical presentation of SLE pericarditis is similar to the classic presentation of acute pericarditis, with typical precordial or substernal pleuritic pain with positional variation of pain [6]. The electrocardiogram (ECG) classically reveals diffuse ST elevation with peaked T-waves, though more commonly shows non-specific T-wave changes or transient ST changes [6]. Recurrence after the first episode is not uncommon and is seen in 15–30% of cases [16,17]. Cardiac tamponade is rather uncommon, but 2% of them may develop tamponade/constrictive pericarditis [8,15].

### 1.3. Myocarditis

Myocarditis is a rare but potentially fatal manifestation of SLE [18]. It is usually subclinical, but 5–10% of all SLE patients develop symptomatic myocarditis [19]. ECG abnormalities vary from non-specific ST-T changes to conduction defects and/or supra or ventricular tachycardia [20] with or without concurrent heart failure [21].

According to the results of the LUMINA study, myocarditis develops early in the course of SLE patients of African American ethnicity and with a higher systemic lupus activity measure-revised (SLAM-R) score at diagnosis. Myocarditis was not associated with higher disease activity over time, but probably had some impact on damage accrual. Patients with myocarditis presented higher mortality, after 5 yrs of disease duration, probably due to the higher damage accrual. It seems that myocarditis in SLE has both intermediate and long-term consequences [22].

### 1.4. Epicardial Coronary Artery Disease (ECAD)

SLE is related to a higher risk of CVD, with an increase of up to 50 times in young people. Furthermore, 30% of deaths are attributed to ECAD. The risk of developing coronary artery disease (CAD) in SLE is related to both traditional CV risk factors and disease-specific factors, such as degree of activity, auto-antibodies, organ damage, and treatment. Accelerated atherosclerosis is the main contributor to ECAD. Clinical presentation ranges from atypical chest pain to acute myocardial infarction and/or sudden cardiac death [23]. A retrospective study of SLE patients who underwent coronary artery angiography showed a high incidence of ECAD. Therefore, we should note that the SLE patients with ECAD showed a similar severity of CAD compared to the controls, despite having less than half the incidence of diabetes mellitus and being 20 years younger. This supports that ECAD in SLE is independent of traditional risk factors [24].

Inflammation and infection in SLE patients induce important alterations in lipid profile, which may initially suppress inflammation/infection, but in the long term may lead to an increased risk of atherosclerosis. The most common lipid changes in SLE include (a) an increase in triglycerides, due to both an increase in hepatic VLDL production/secretion and a decrease in the clearance of triglyceride-rich lipoproteins, and (b) a decrease in serum HDL, with the mechanism of this phenomenon remaining uncertain. With inflammation, there is also a consistent increase in lipoprotein (a) levels, due to increased apolipoprotein (a) synthesis. The prevalence of small dense LDL is increased due to the exchange of triglycerides from triglyceride-rich lipoproteins to LDL, followed by triglyceride hydrolysis. Furthermore, the greater the severity of the inflammatory disease, the more often these abnormalities in lipids and lipoproteins are observed. Treatment of the underlying disease with reduction of the inflammatory process leads to normalization of the lipid profile. The standard risk calculators for predicting CVD (ACC/AHA, Framingham, SCORE, etc.) underestimate the risk in patients with inflammation. It has been recommended to increase the calculated risk by approximately 50% in patients with severe underlying inflammatory disorders. The treatment of lipid disorders in SLE patients is similar to patients without inflammatory disorders. Of note, statins, fibrates, and fish oil have anti-inflammatory properties and present beneficial effects on some of these inflammatory disorders [25].

Lastly, we should note that spontaneous coronary artery dissection (SCAD), although it is a rare phenomenon, can present as an acute coronary syndrome (ACS) and sudden cardiac death, especially in young women [26].

### 1.5. Microvascular Coronary Artery Disease (MCAD)

Systemic inflammation is an important risk factor for coronary microvascular dysfunction (CMD). SLE patients with cardiac symptoms and unobstructed epicardial coronary arteries have a high prevalence of coronary vasomotor abnormalities. In comparison with symptomatic matched controls, SLE patients have a more severe reduction in myocardial flow reserve (MFR), which cannot be explained by the traditional CVD risk factors or atherosclerotic burden [27]. A 5-year follow-up study of SLE patients with chest pain (CP), evaluated at baseline and follow-up, showed that the majority had persistent CP, and nearly one half had similar or worse myocardial perfusion consistent with CMD without obstructive CAD [28,29].

### 1.6. Aortic Disease

Aortic aneurysm and dissection are rare complications of SLE. The incidence, etiology, risk factors, and outcomes of this entity were largely unknown. A total of 36 articles reporting a single case or case series involving 40 patients were evaluated. The patients showed an absolute female dominance at a mean aneurysm age of 44.6 years. Steroid use had a duration of 13.3 ± 9.4 years prior to admission for management of aortic aneurysm/dissection. Aortic aneurysm occurred more commonly in the abdominal than in other segments of the aorta, whereas aortic dissection did not show any location predilection.

Hypertension, long-term steroid use, and aortic pathological changes related to SLE seemed to be predominant risk factors for the occurrence of aortic aneurysm and dissection. Upon diagnosis, a surgical, interventional, or hybrid treatment should be performed to prevent severe complications and/or sudden deaths [30].

## 2. Methodology

This review aimed to comprehensively cluster and summarize available data on laboratory parameters and imaging modalities for CVD risk stratification in patients with SLE. A comprehensive literature review was conducted in the PubMed database using the key terms cardiovascular disease and SLE, and imaging or auto-antibodies, or genetic factors. The relevant original and review articles that have been published in peer-reviewed journals were included in the list.

## 3. Results

The risk stratification of SLE patients was based on noninvasive imaging modalities, genetic factors, and disease characteristics.

### 3.1. Imaging Modalities for Assessment of Cardiovascular Involvement

The more commonly used noninvasive imaging modalities for the CV assessment in SLE are described below.

#### 3.1.1. Echocardiography

Echocardiography is the first noninvasive imaging modality used for the assessment of SLE, as it is bedside, widely available, low-cost, and has no radiation exposure. According to an echocardiographic study, cardiac involvement was found in 52% of SLE patients. Of them, 38% had pericardial effusion, 48% valvular abnormalities, and 12.5% myocarditis. Increased pulmonary arterial hypertension (PAH) was reported in 10%. The significant correlation between SLE disease duration and cardiac abnormalities supports the routine echocardiographic evaluation of SLE patients, as cardiac involvement is usually clinically silent [31].

Recently, Global Longitudinal Strain (GLS) via speckle tracking echocardiography has been used to detect early, preclinical cardiac involvement in SLE. It is important to note that GLS reveals cardiac abnormalities in SLE patients, with normal left ventricular ejection fraction (LVEF) indicating that GLS changes can potentially be used as an early imaging marker of cardiac dysfunction. However, GLS cannot provide information about cardiac involvement etiology (oedema, ischemia, fibrosis) [32]. Furthermore, GLS impairment and LV dyssynchrony were identified in treatment-naïve patients with new-onset SLE and normal LVEF [33]. The same was also found from the meta-analysis of tracking echocardiography in SLE patients [34].

#### 3.1.2. Cardiac Computed Tomography (CCT)

Cardiac Computed Tomography (CCT) uses various approaches to provide reliable and reproducible information regarding coronary anatomy and function. These approaches include:

- Coronary artery calcium scoring.

It has been used to identify plaque burden in patients with coronary artery calcification (CAC) and is obtained without using contrast agents. It provides quantitative information regarding CAC and facilitates the assessment of CV risk. A calcium score of zero Hounsfield units indicates a lack of calcium in the coronary arteries and represents a strong negative risk predictor for CAD. Early screening of SLE patients with CT calcium scoring could facilitate the early diagnosis/treatment, leading to the prevention of premature coronary atherosclerosis and future cardiac events. The Society of Cardiovascular Imaging has endorsed the application of a baseline calcium artery score with a repeat progression scan in 3–5 years [35].

- Computed Tomography Coronary Angiography (CTCA).

CAC is the expression of an advanced stage of atherosclerosis, while noncalcified coronary atherosclerotic plaques (NCP) are more prone to trigger acute coronary syndrome (ACS). This reason led to Computed Tomography Coronary Angiography (CTCA).

NCPs are more likely to present rapid progression compared to calcified coronary plaque (CCP). However, neither SLE disease activity and traditional CVD risk factors nor SLE serology or use of prednisone/hydroxychloroquine can predict progression of NCP over time. Immunosuppressive drugs such as mycophenolate mofetil and methotrexate have a protective effect on the progression of NCP [36]. Furthermore, a recent systematic review and meta-analysis showed that, in SLE patients, subclinical CAD, assessed by calcium score and CTCA, is more prevalent compared with controls [37]. A CTCA study of coronary plaque volume in patients with rheumatoid arthritis (RA) and SLE showed that the coronary plaque volume was similar in RA and SLE patients at baseline, but the progression was greater in SLE, which may explain the higher CVD risk in this disease [38].

Radiation dose and nephrotoxic effect of CT-contrast agent represent the major limitations of the routine use of CTCA. However, CTCA radiation dose has progressively decreased, except in coronary artery bypass grafting (CABG) and arrhythmia cases [39].

- Fractional flow reserve (FFR).

Fractional flow reserve (FFR) is considered to be one of the diagnostic methods for identifying patients who will benefit from revascularization [40,41,42,43]. FFR is considered to be a class IIa recommendation for treatment decision making [44,45].

Many multicenter studies have demonstrated the high diagnostic impact of CCTA to identify CAD stenosis [46,47,48]. However, coronary stenosis severity does not always correlate well with the functional severity of CAD, detected by invasive FFR. In the FAME study [49], it was found that 20% showed FFR > 0.80 among 70–90% of severe stenoses, identified by interventional coronary angiography (ICA), and 65% had FFR > 0.80 among 50–69% of moderate ICA stenosis. Although many studies have shown the prognostic value of anatomical stenosis by CCTA [50,51], this misclassification may influence the risk stratification of patients with suspected CAD.

Currently, the CT-derived FFR reveals the “functional significance” of coronary lesions under hyperemic conditions. This can be estimated by computational flow modeling without the use of stress factors such as adenosine. This noninvasive method may be an alternative to ICA with FFR [52], but similar studies in SLE are missing.

#### 3.1.3. Nuclear Techniques

The main nuclear techniques are the following:

- Single-photon emission computed tomography (SPECT)

A SPECT study of SLE patients showed the high presence of premature, accelerated atherosclerosis in young SLE patients. According to this study, younger SLE patients with poor disease control, higher SLEDAI score, less aggressive treatment, increased high-sensitivity C-reactive protein (hsCRP) values, and increased pre-coagulant tendency should undergo screening for myocardial perfusion abnormalities using SPECT [53].

- Cardiac positron emission tomography (PET)

A study using stress cardiac positron emission tomography-computed tomography (PET-CT), applied in SLE patients with cardiac symptoms, but without obstructive CAD and/or systolic dysfunction, showed that they have a high prevalence of coronary vasomotor abnormalities. In comparison with symptomatic matched controls, SLE patients had a more severe reduction in myocardial flow reserve (MFR) that cannot be explained by common CVD factors or atherosclerotic burden [27].

PET is superior to SPECT for the assessment of CVD in SLE, as it can identify both myocardial ischemia and inflammation with less radiation than SPECT. The high diagnostic accuracy and the low radiation support its wider application. PET also allows for measurement of myocardial blood flow (MBF) and myocardial flow reserve (MFR), which is mandatory to clarify complex clinical scenarios [54].

#### 3.1.4. Cardiovascular Magnetic Resonance (CMR)

**Figure 1:**
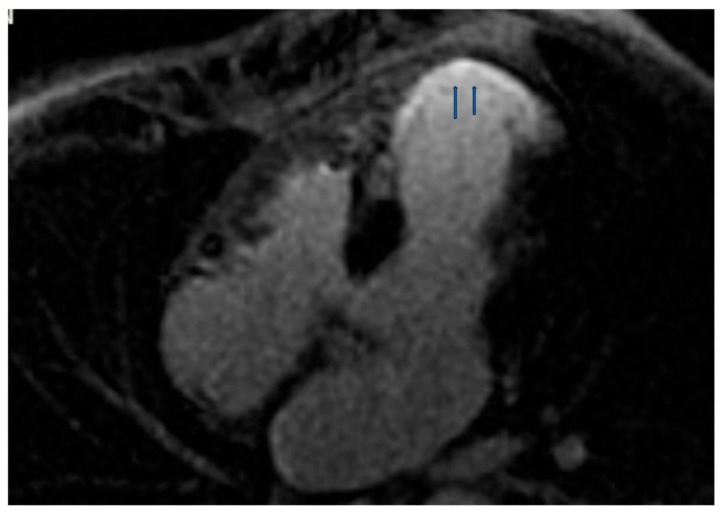
Extensive transmural myocardial infarction (arrow), due to Left anterior descending coronary artery (LAD) occlusion [Source: author’s archives (S.I.M.)].

CMR is the ideal tool to evaluate noninvasively and without radiation cardiac function, perfusion, and fibrosis in the same examination. At the 5-year follow-up of SLE patients with CP, using stress CMR, the majority had persistent CP, and nearly one half had similar or worse myocardial perfusion consistent with CMD without obstructive CAD [28]. In another study, a 44% prevalence of abnormal stress myocardial perfusion was identified using CMR in the absence of obstructive CAD in SLE patients with angina [29]. Compared with controls, reduced myocardial perfusion rate index (MPRI) was observed in SLE patients, with SLE diagnosis being a significant predictor of an abnormal MPRI. These findings are consistent with the hypothesis that anginal CP in SLE without obstructive CAD is due to microvascular coronary artery dysfunction [29]. Furthermore, reduced MPRI and late gadolinium enhancement (LGE) presence were common in asymptomatic patients with APS and SLE with APS, independently of any APS-related and classic CVD risk factors, or coronary angiography findings in cases with LGE [55]. Lastly, a CMR study in SLE showed abnormal findings in 43%, including LGE (Figure 1), stress perfusion deficits, and pericardial effusion [56]. Patients with non-ischemic LGE more often had microalbuminuria, while patients with stress perfusion deficits had a history of hypertension. However, no correlation between clinical symptoms and CMR results was identified [56]. Furthermore, CMR in SLE patients with atypical cardiac symptoms/signs and normal echocardiographic findings can assess occult cardiac lesions, including myocarditis, myocardial infarction, and vasculitis that may have an impact on both anti-rheumatic and cardiac treatment [57].

**Figure 2:**
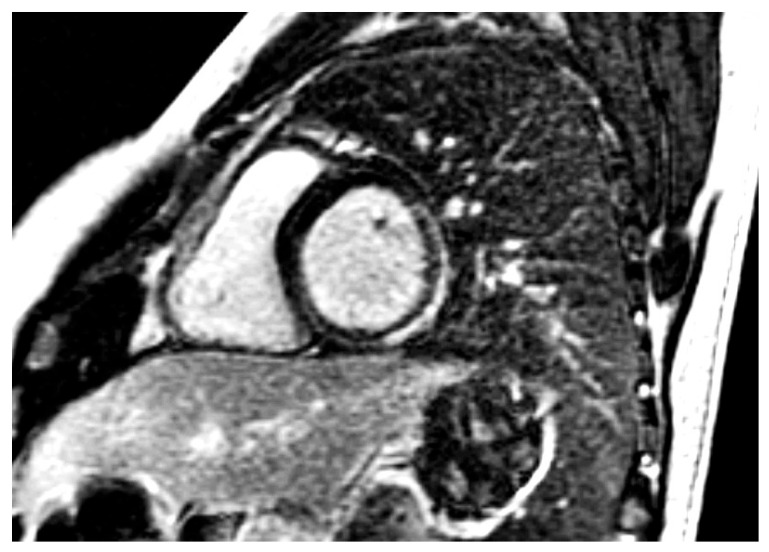
Short axis inversion recovery showing epicardial LGE in the lateral wall of LV [Source: author’s archives (S.I.M.)].

The presence of myocardial inflammation is another crucial point in the evaluation of SLE. CMR is the ideal tool for both diagnosis and treatment follow-up of myo-pericardial inflammation (Figure 2), as it can detect a preclinical inflammatory process. Myocardial inflammation was detected in clinically overt SLE [58] and in treatment-naïve SLE patients at diagnosis [59]. Lastly, increased myocardial T2-mapping values were identified in SLE patients, due to subclinical myocardial oedema, suggesting that even in SLE patients with inactive systemic disease and normal cardiac function, low-grade myocardial inflammation can be detected using this technique [60].

If the noninvasive evaluation suggests the presence of ischemic heart disease, the X-ray coronary angiography verifies the diagnosis and guides further therapeutic approach (medical treatment, angioplasty, coronary artery bypass surgery, etc.).

### 3.2. Genetic Factors and CVD in SLE

Genetic factors are critical in CVD pathogenesis during SLE. Therefore, currently, genetic risk scores have been developed and have become commercially available. Evidence supports the presence of a considerable genetic component in patients with SLE susceptible to the development of CAD. According to the results of a meta-analysis, a single-nucleotide polymorphism (SNP) rs925994 located in the interferon regulatory factor-8 (IRF8) gene exhibited a strong association with CAD, presence of carotid plaques, and increased intima-media thickness in SLE [61].

An IL19 risk allele, rs17581834(T), was associated with both stroke and myocardial infarction in patients with SLE, but this was not lupus-specific, as it was also present in patients with RA. In patients with SLE, the rs799454(G) SRP54-AS1 allele was associated with stroke/transient ischemic attack [62]. However, neither the IL19 risk allele nor the SRP54-AS1 risk alleles were associated with SLE disease per se or the presence of carotid plaques. Based on these data, it was suggested that additional mechanisms from those observed in the general population may account for the development of CVD in SLE.

The HLA-DRB1*13 allele of the major histocompatibility complex (MHC) region was associated with the development of all kinds of vascular events, including ischemic heart disease, ischemic cerebrovascular disease, and venous thromboembolism. It was also associated with anti-β2-glycoprotein-1 IgG, anticardiolipin IgG, and anti-prothrombin IgG [63]. Another MHC allele, the HLA-DRB1*04, was also associated with pro-thrombotic auto-antibodies such as anti-β2-glycoprotein-1 IgG, anticardiolipin IgM and IgG, anti-prothrombin IgG, and with a positive lupus anticoagulant (LA) test. SLE patients with both alleles HLA-DRB1*04/*13 MHC demonstrated a higher risk of vascular events.

A bioinformatic analysis and machine learning algorithms approach illustrated that 5 hub genes (SPI1, MMP9, C1QA, CX3CR1, MNDA) could predict the risk of atherosclerosis in SLE [64]. Mendelian randomization (MR) analyses were performed to assess whether a potential causal association existed between genetic susceptibility to the development of both SLE and CVD [65,66,67]. However, data on the lupus genetic risk are inconclusive, as the results of different studies are often conflicting.

Genetic factors that are associated with specific vascular events are summarized in Table 1.

### 3.3. Auto-Antibodies, Disease Manifestations, Medications, and CVD in SLE

#### 3.3.1. Auto-Antibodies

The presence of antiphospholipid antibodies (aPL) in the serum has been associated apart from morbidity during pregnancy, with vascular thrombotic effects. It has therefore been proposed that SLE patients with positive serum aPL warrant surveillance for future atherosclerotic CVD. To this end, a multicenter prospective study enrolling more than 1500 patients with SLE in China disclosed that 106 patients developed CVD during a 4.5-year follow-up [71]. Ninety-two out of these 106 patients were aPL positive. More specifically, a positive LA test was associated with a 5 times higher risk of CVD, and anticardiolipin (IgM and IgG) with a 2 times higher CVD risk. Of note, treatment with antiplatelet or oral anticoagulants was associated with a reduced CVD risk in aPL (+) lupus patients. Furthermore, the 10-year risk of incident CVD events was found to be higher in SLE patients, compared to healthy controls, and was associated with persistent triple aPL positivity [72]. Additionally, vascular events were significantly increased in patients with double/triple aPL positivity compared to those with single aPL positivity during early SLE [73]. High titers of IgG aPL positivity did not increase the risk of the development of vascular events. However, the IgG isotype was associated with a higher risk of CVD compared to the IgM isotype for both anti-β2GPI and anticardiolipin (aCL) antibodies [74].

The adjusted Global Antiphospholipid Syndrome Score (aGAPSS), which incorporates independent CVD risk factors along with the serum aPL profile, has been proposed as a tool in predicting CVD risk in patients with SLE [75]. The aGAPSS is calculated by adding points corresponding to the risk factors: 3 for hyperlipidemia, 1 for arterial hypertension, 5 for positive aCL antibodies, 4 for anti-β2GPI antibodies, and 4 for a positive LA test. A significant association was noticed between the occurrence of cerebrovascular and coronary events and high values of aGAPSS. A strong association is observed between circulating aCL IgG antibodies and non-thrombotic cardiac manifestations, including CAD, aortic/mitral/tricuspid regurgitation, mitral valve prolapse, and cardiomyopathy [76]. An aGAPSS score > 8.5 was predictive of valve disease or CAD, while aGAPSS scores > 9.5 were predictive of cardiomyopathies.

Another study reported that patients with SLE positive for anti-dsDNA auto-antibodies exhibited endothelial dysfunction, dyslipidemia, accelerated atherosclerosis, and aberrant innate immune cell activation leading to an increased CVD risk [77]. More specifically, anti-dsDNA antibodies with a distinct gene and protein expression profile promote neutrophil extracellular trap (NET) formation and the apoptosis of monocytes. They also regulate the expression of inflammatory and thrombotic mediators and induce endothelial cell activation. Finally, anti-dsDNA antibodies mediate myeloid cell activation through FcR-mediated binding mechanisms. The association between anti-dsDNA auto-antibodies and CVD was shown to be independent of other risk factors, suggesting a direct impact of anti-dsDNA positivity.

A possible association between circulating anti-Ro auto-antibodies and myocarditis or heart conduction defects in patients with SLE has also been reported [78]. Anti-Ro antibodies reportedly interact with heart ion channels, leading to conduction abnormalities, QTc prolongation, and arrhythmias [79].

Nevertheless, cardiac screening is not recommended for anti-Ro/SSA (+) lupus patients, as no significant changes were observed in ECG, 24-h Holter monitoring, or echocardiographic measurements [80]. Anti-CL and anti-Ro circulating auto-antibodies were also associated with plaque progression in another SLE study from the UK [81].

#### 3.3.2. Lupus Clinical Manifestations

Associations between CVD and overall disease activity or disease-associated damage have also been attempted. A prospective study showed that the development of CVD events was independently associated with increased triglyceride levels, previous cyclophosphamide treatment, and the Systemic Lupus International Collaborating Clinics damage index (SDI) score at baseline. A meta-analysis reported that predictors of CVD in SLE included traditional risk factors, such as male gender, hyperlipidemia, family history of CVD, hypertension, and non-traditional factors such as the presence of aCL auto-antibodies [82]. Another review identified SLE-specific CVD risk factors, such as long-term disease duration, high disease activity assessed using SLEDAI-2K, and organ damage [83].

Lupus nephritis (LN) has also been considered to be a potential risk factor for the development of CVD. The results of a meta-analysis illustrated that the risk of CVD was double in patients with LN, compared to SLE patients without [84]. The proliferative classes of LN, such as class III and class III/V LN, have a stronger association with CAD [85].

Studies have disclosed the racial variation in the risk of CVD events among patients with SLE. The risk of CVD events was found to be increased in Blacks compared to Whites. The risk of MI was lower in Hispanics and Asians, whereas the risk of stroke was higher in Blacks and Hispanics [86]. Furthermore, the highest frequency of CVD occurred at 2–11 years after SLE diagnosis, and this was mainly observed in Black patients. Additionally, a higher incidence of MI and stroke was observed in Black women < 55 years of age [87]. The same study reported a strong association between discoid rash in the first year of SLE diagnosis and CVD, an effect that was race-independent. However, we should note that discoid lupus rashes are much less common in non-African American lupus patients.

#### 3.3.3. Medications

Certain medications may also contribute to the development of CVD in SLE. Traditionally, treatment with corticosteroids has been considered to be a risk factor for CVD. The cumulative dose of corticosteroids has not been associated with CVE rates [88]. However, patients on current treatment with ≥20 mg/d of prednisone exhibited a substantially increased risk of CVD, which means that the current dose of corticosteroids might be a stronger predictor of CVD, compared to the cumulative dose.

Hydroxychloroquine (HCQ) is currently recommended for all patients with SLE, unless contraindicated. Cardiac toxicity induced by HCQ is rare, manifesting mostly as conduction system abnormalities [89]. This HCQ-induced cardiomyopathy must be documented with a biopsy. In contrast to the rarity of HCQ-induced cardiotoxicity, antimalarial treatment in patients with SLE has a clearly established cardioprotective and vasculo-protective effect. The LUMINA study disclosed the CVD-protective role of currently employed HCQ. However, remote HCQ treatment was not associated with ongoing CVD protection, underscoring the need for continuous HCQ treatment in SLE [90]. It seems that SLE clinical manifestations, auto-immune profile, and anti-rheumatic medications play a vital role in CVD development in SLE.

A statistically not significant reduced CVD risk was observed in SLE patient-initiated treatment with mycophenolate mofetil (MMF) compared to those initiated treatment with cyclophosphamide (CYC) or azathioprine (AZA). However, a 12-month intention-to-treat analysis reported that the risk of a first CVD event was significantly lower in patients treated with MMF compared to those treated with AZA [91]. On the other hand, a study showed that belimumab, the first approved biologic treatment in patients with SLE, improved the HDL atheroprotective role and regulated the HDL lipidomic signature in patients with SLE [92]. However, the results of this study cannot address the possible impact of belimumab treatment on the development of CVD in patients with SLE, due to the short-term follow-up of the study.

#### 3.3.4. Inflammatory Mediators

Among inflammatory mediators associated with atherosclerosis, CRP might be a potent predictor for CVD in patients with SLE. Furthermore, hsCRP measurement might be a useful marker of increased IMT and subclinical atherosclerosis in patients with SLE. IL-6 is a proinflammatory cytokine regulating the release of fatty acids, contributing to an increased cardiovascular mortality and worse prognosis in the general population. Soluble vascular cellular adhesion molecule (sVCAM)-1 was reportedly increased only in patients with SLE and CVD. Patients with SLE demonstrated increased carotid IMT and vascular endothelial growth factor (VEGF) concentrations. Tumor necrosis factor-α (TNF-α) may also be an important factor for the development of CVD in patients with SLE [93,94].

## 4. Traditional Risk Factors

Classical risk factors that may contribute to the development of accelerated atherosclerosis in patients with SLE include non-modifiable ones such as older age, male gender, and family history of CVD, as well as modifiable factors such as arterial hypertension, dyslipidemia, mellitus diabetes, cigarette smoking, and increased plasma homocysteine concentrations [95]. However, analyses performed after adjustments for the traditional risk factors attribute a less important role to them for the increased CVD risk in patients with SLE.

## 5. Discussion

Disease activity, auto-antibodies, and genetic indices represent the “clinico-laboratory background” of SLE patients. However, it is the addition of cardiovascular imaging that can detect the type and activity of cardiovascular involvement. In contrast to what was traditionally believed in the past, imaging evaluation is not only a radiologic field, but also a clinical tool that clinicians should be able to understand and interpret. Taking into consideration laboratory, genetic, and imaging results, we propose an algorithm for SLE CVD risk stratification.

**Figure 3:**
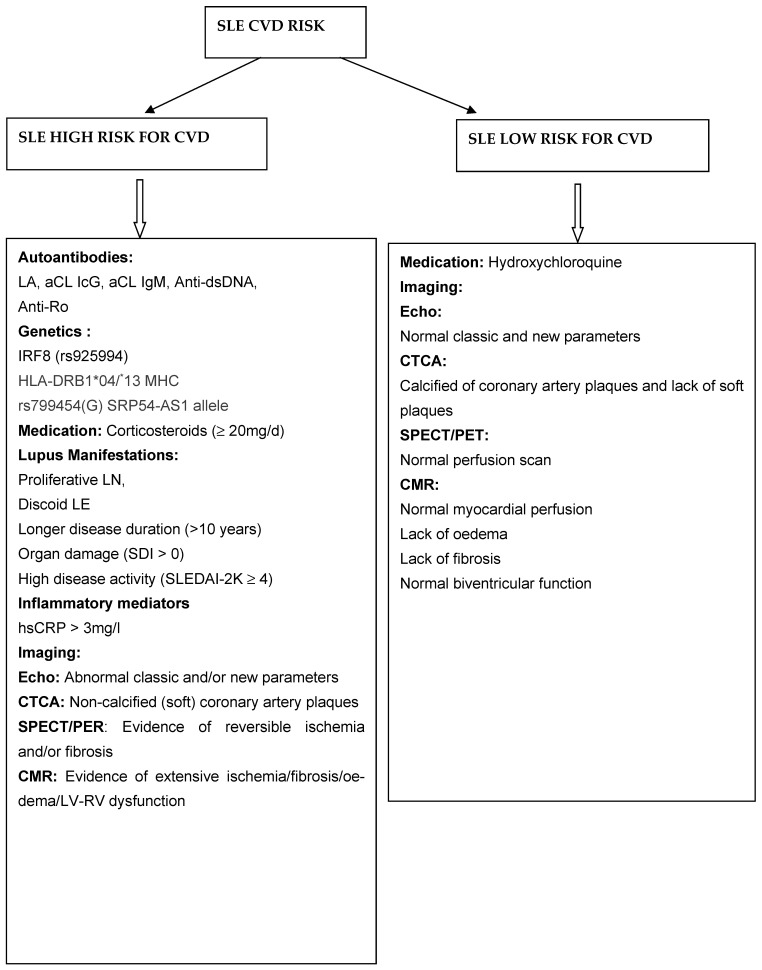
Algorithm to predict CVD severity in SLE.

We included clinical, genetic, and imaging characteristics in the following Figure 3, where we present the most relevant information regarding CVD in SLE in order to provide a clinically useful algorithm regarding CVD severity in SLE.

## Figures and Tables

**Table 1:** Genetic factors associated with specific vascular events.

SNP rs10181656(G) in the STAT4 gene [68]	Ischemic cerebrovascular events
Homozygosity for mannose-binding lectin [69]	Arterial thrombotic events
GT20 allele of the CRP gene [70]	Vascular arterial events in African Americans and Hispanics

SNP: single-nucleotide polymorphism, STAT4: signal transducer and activator of transcription factor 4, CRP: C-reactive protein.

## Data Availability

No new data were created or analyzed in this study. Data sharing is not applicable to this article.

## Abbreviations

The following abbreviations are used in this manuscript:

SLE	systemic lupus erythematosus
RA	rheumatoid arthritis
CVD	cardiovascular disease
CAD	coronary artery disease
NBTE	nonbacterial thrombotic endocarditis
APS	antiphospholipid syndrome
ECAD	epicardial coronary artery disease
SCAD	spontaneous coronary artery dissection
ACS	acute coronary syndrome
MCAD	microvascular coronary artery disease
CP	chest pain
PAH	pulmonary arterial hypertension
GLS	global longitudinal strain
LVEF	left ventricular ejection fraction
CCT	cardiac computed tomography
CAC	coronary artery calcification
CTCA	computed tomography coronary angiography
NCP	noncalcified coronary atherosclerotic plaque
ACS	acute coronary angiography
CCP	calcified coronary plaque
FFR	fractional flow reserve
ICA	interventional coronary angiography
SPECT	single-photon emission computed tomography
PET	positron emission tomography
MFR	myocardial flow reserve
MBF	myocardial blood flow
CMR	cardiovascular magnetic resonance
MPRI	myocardial perfusion rate index
SNP	single-nucleotide polymorphism
IRF8	interferon regulatory factor-8
MHC	major histocompatibility complex
MR	mendelian randomization
aCL	anticardiolipin
aPL	antiphospholipid antibodies
aGAPSS	adjusted global antiphospholipid syndrome score
ECG	electrocardiogram
SLEDAI-2K	systemic lupus erythematosus disease activity index-2000
SDI	systemic lupus international collaborating clinics damage index
LN	lupus nephritis
HCQ	hydroxychloroquine
MI	myocardial infarction
dsDNA	double-stranded DNA
RNP	ribonucleoprotein

## References

[1] Miner J.J., Kim A.H. (2014). Cardiac manifestations of systemic lupus erythematosus. Rheum. Dis. Clin. N. Am..

[2] Nor M.A., Ogedegbe O.J., Barbarawi A., Ali A.I., Sheikh I.M., Yussuf F.M., Adam S.M., Hassan O.A., Tabowei G., Jimoh A. (2023). Systemic Lupus Erythematosus and Cardiovascular Diseases: A Systematic Review. Cureus.

[3] Tselios K., Urowitz M.B. (2017). Cardiovascular and Pulmonary Manifestations of Systemic Lupus Erythematosus. Curr. Rheumatol. Rev..

[4] Cervera R., Khamashta M.A., Font J., Sebastiani G.D., Gil A., Lavilla P., Mejía J.C., Aydintug A.O., Chwalinska-Sadowska H., de Ramón E. (2003). Morbidity and mortality in systemic lupus erythematosus during a 10-year period: A comparison of early and late manifestations in a cohort of 1000 patients. Medicine.

[5] Ryu S., Fu W., Petri M.A. (2017). Associates and predictors of pleurisy or pericarditis in SLE. Lupus Sci. Med..

[6] Tincani A., Rebaioli C.B., Taglietti M., Shoenfeld Y. (2006). Heart involvement in systemic lupus erythematosus, anti-phospholipid syndrome and neonatal lupus. Rheumatology.

[7] Zhao J., Bai W., Zhu P., Zhang X., Liu S., Wu L., Ma L., Bi L., Zuo X., Sun L. (2016). Chinese SLE Treatment and Research group (CSTAR) registry VII: Prevalence and clinical significance of serositis in Chinese patients with systemic lupus erythematosus. Lupus.

[8] Moder K.G., Miller T.D., Tazelaar H.D. (1999). Cardiac involvement in systemic lupus erythematosus. Mayo Clin. Proc..

[9] Feng J.B., Ni J.D., Yao X., Pan H.F., Li X.P., Xu J.H., Pan F.M., Xu S.Q., Ye D.Q. (2010). Gender and age influence on clinical and laboratory features in Chinese patients with systemic lupus erythematosus: 1790 cases. Rheumatol. Int..

[10] Mittoo S., Gelber A.C., Hitchon C.A., Silverman E.D., Pope J.E., Fortin P.R., Pineau C., Smith C.D., Arbillaga H., Gladman D.D. (2010). Clinical and serologic factors associated with lupus pleuritis. J. Rheumatol..

[11] Tang X., Huang Y., Deng W., Tang L., Weng W., Zhang X. (2010). Clinical and serologic correlations and autoantibody clusters in systemic lupus erythematosus: A retrospective review of 917 patients in South China. Medicine.

[12] Yasuma M., Takasaki Y., Matsumoto K., Kodama A., Hashimoto H., Hirose S. (1990). Clinical significance of IgG anti-Sm antibodies in patients with systemic lupus erythematosus. J. Rheumatol..

[13] Jurencák R., Fritzler M., Tyrrell P., Hiraki L., Benseler S., Silverman E. (2009). Autoantibodies in pediatric systemic lupus erythematosus: Ethnic grouping, cluster analysis, and clinical correlations. J. Rheumatol..

[14] Doria A., Iaccarino L., Sarzi-Puttini P., Atzeni F., Turriel M., Petri M. (2005). Cardiac involvement in systemic lupus erythematosus. Lupus.

[15] Kahl L.E. (1992). The spectrum of pericardial tamponade in systemic lupus erythematosus. Report of ten patients. Arthritis Rheum..

[16] Rosenbaum E., Krebs E., Cohen M., Tiliakos A., Derk C.T. (2009). The spectrum of clinical manifestations, outcome and treatment of pericardial tamponade in patients with systemic lupus erythematosus: A retrospective study and literature review. Lupus.

[17] Imazio M., Spodick D.H., Brucato A., Trinchero R., Adler Y. (2010). Controversial issues in the management of pericardial diseases. Circulation.

[18] Jacobsen S., Petersen J., Ullman S., Junker P., Voss A., Rasmussen J.M., Tarp U., Poulsen L.H., van Overeem Hansen G., Skaarup B. (1998). A multicentre study of 513 Danish patients with systemic lupus erythematosus. II. Disease mortality and clinical factors of prognostic value. Clin. Rheumatol..

[19] Law W.G., Thong B.Y., Lian T.Y., Kong K.O., Chng H.H. (2005). Acute lupus myocarditis: Clinical features and outcome of an oriental case series. Lupus.

[20] Wijetunga M., Rockson S. (2002). Myocarditis in systemic lupus erythematosus. Am. J. Med..

[21] Ashour A.A., Mansour S., Talal Basrak M., Altermanini M., Sawaf B., Atta M.A., Habib M.B. (2023). Case report: Severe sinus tachycardia as a leading manifestation of systemic lupus erythematosus flare. Front. Med..

[22] Apte M., McGwin G., Vilá L.M., Kaslow R.A., Alarcón G.S., Reveille J.D., LUMINA Study Group (2008). Associated factors and impact of myocarditis in patients with SLE from LUMINA, a multiethnic US cohort (LV). Rheumatology.

[23] Melano-Carranza E., Zambrano-Zambrano A., Valle-Uitzil W., Ezquerra-Osorio A., Rodriguez-Méndez A., Larios-Lara J.H., Baeza L., Pimentel-Esparza J.A., Cervantes-Nieto J.A., Fuentes Mendoza J.A. (2023). Coronary Artery Disease in Systemic Lupus Erythematosus: What Do the Facts Say?. Cureus.

[24] Kaul M.S., Rao S.V., Shaw L.K., Honeycutt E., Ardoin S.P., St Clair E.W. (2013). Association of systemic lupus erythematosus with angiographically defined coronary artery disease: A retrospective cohort study. Arthritis Care Res..

[25] Feingold K.R., Grunfeld C., Feingold K.R., Anawalt B., Blackman M.R., Boyce A., Chrousos G., Corpas E., de Herder W.W., Dhatariya K., Hofland J., Kalra S. (2000). The Effect of Inflammation and Infection on Lipids and Lipoproteins. Endotext.

[26] Patel R., Patel R., Rahming H., Tian J., Kandov R. (2023). Spontaneous Coronary Artery Disease (SCAD) in a Patient With Systemic Lupus Erythematosus (SLE). Cureus.

[27] Weber B.N., Stevens E., Barrett L., Bay C., Sinnette C., Brown J.M., Divakaran S., Bibbo C., Hainer J., Dorbala S. (2021). Coronary Microvascular Dysfunction in Systemic Lupus Erythematosus. J. Am. Heart Assoc..

[28] Sandhu V.K., Wei J., Thomson L.E.J., Berman D.S., Schapira J., Wallace D., Weisman M.H., Bairey Merz C.N., Ishimori M.L. (2020). Five-Year Follow-Up of Coronary Microvascular Dysfunction and Coronary Artery Disease in Systemic Lupus Erythematosus: Results From a Community-Based Lupus Cohort. Arthritis Care Res..

[29] Ishimori M.L., Martin R., Berman D.S., Goykhman P., Shaw L.J., Shufelt C., Slomka P.J., Thomson L.E., Schapira J., Yang Y. (2011). Myocardial ischemia in the absence of obstructive coronary artery disease in systemic lupus erythematosus. JACC Cardiovasc. Imaging.

[30] Yuan S.M. (2019). Aortic aneurysm and dissection in systemic lupus erythematosus. Z. Rheumatol..

[31] Drissa M., Helali S., Amani F., Chebbi M., Mechri M., Drissa H. (2019). Echocardiographic characteristics of cardiac involvement in Systemic lupus erythematosus. Tunis. Med..

[32] Nikdoust F., Bolouri E., Tabatabaei S.A., Goudarzvand M., Faezi S.T. (2018). Early diagnosis of cardiac involvement in systemic lupus erythematosus via global longitudinal strain (GLS) by speckle tracking echocardiography. J. Cardiovasc. Thorac. Res..

[33] Luo T., Wang Z., Chen Z., Yu E., Fang C. (2021). Layer-specific strain and dyssynchrony index alteration in new-onset systemic lupus erythematosus patients without cardiac symptoms. Quant. Imaging Med. Surg..

[34] Di Minno M.N.D., Forte F., Tufano A., Buonauro A., Rossi F.W., De Paulis A., Galderisi M. (2020). Speckle tracking echocardiography in patients with systemic lupus erythematosus: A meta-analysis. Eur. J. Intern. Med..

[35] Wu M., Mirkin S., Nagy S., McPhail M.N., Demory Beckler M., Kesselman M.M. (2023). Computed Tomography (CT) Calcium Scoring in Primary Prevention of Acute Coronary Syndrome and Future Cardiac Events in Patients With Systemic Lupus Erythematosus. Cureus.

[36] Khan A., Arbab-Zadeh A., Kiani A.N., Magder L.S., Petri M. (2017). Progression of noncalcified and calcified coronary plaque by CT angiography in SLE. Rheumatol. Int..

[37] Mendoza-Pinto C., Munguía-Realpzo P., García-Carrasco M., Godinez-Bolaños K., Rojas-Villarraga A., Morales-Etchegaray I., Ayón-Aguilar J., Méndez-Martínez S., Cervera R. (2022). Asymptomatic coronary artery disease assessed by coronary computed tomography in patients with systemic lupus erythematosus: A systematic review and meta-analysis. Eur. J. Intern. Med..

[38] Moore J., Lakshmanan S., Manubolu V.S., Kinninger A., Stojan G., Goldman D.W., Petri M., Budoff M., Karpouzas G.A. (2023). Coronary plaque progression is greater in systemic lupus erythematosus than rheumatoid arthritis. Coron. Artery Dis..

[39] Lin Z.X., Zhou C.S., Schoepf U.J., Eid M., Duguay T.M., Greenberg W.T., Luo S., Quan W., Zhou F., Lu G.M. (2019). Coronary CT angiography radiation dose trends: A 10-year analysis to develop institutional diagnostic reference levels. Eur. J. Radiol..

[40] Pijls N.H., van Schaardenburgh P., Manoharan G., Boersma E., Bech J.W., van’t Veer M., Bär F., Hoorntje J., Koolen J., Wijns W. (2007). Percutaneous coronary intervention of functionally nonsignificant stenosis: 5-year follow-up of the DEFER Study. J. Am. Coll. Cardiol..

[41] Pijls N.H., Fearon W.F., Tonino P.A., Siebert U., Ikeno F., Bornschein B., van’t Veer M., Klauss V., Manoharan G., Engstrøm T. (2010). Fractional flow reserve versus angiography for guiding percutaneous coronary intervention in patients with multivessel coronary artery disease: 2-year follow-up of the FAME (Fractional Flow Reserve Versus Angiography for Multivessel Evaluation) study. J. Am. Coll. Cardiol..

[42] De Bruyne B., Pijls N.H., Kalesan B., Barbato E., Tonino P.A., Piroth Z., Jagic N., Möbius-Winkler S., Rioufol G., Witt N. (2012). Fractional flow reserve-guided PCI versus medical therapy in stable coronary disease. N. Engl. J. Med..

[43] De Bruyne B., Fearon W.F., Pijls N.H., Barbato E., Tonino P., Piroth Z., Jagic N., Mobius-Winckler S., Rioufol G., Witt N. (2014). Fractional flow reserve-guided PCI for stable coronary artery disease. N. Engl. J. Med..

[44] Windecker S., Kolh P., Alfonso F., Collet J.P., Cremer J., Falk V., Filippatos G., Hamm C., Head S.J., Authors/Task Force Members (2014). 2014 ESC/EACTS Guidelines on myocardial revascularization: The Task Force on Myocardial Revascularization of the European Society of Cardiology (ESC) and the European Association for Cardio-Thoracic Surgery (EACTS) Developed with the special contribution of the European Association of Percutaneous Cardiovascular Interventions (EAPCI). Eur. Hear. J..

[45] Levine G.N., Bates E.R., Blankenship J.C., Bailey S.R., Bittl J.A., Cercek B., Chambers C.E., Ellis S.G., Guyton R.A., Hollenberg S.M. (2011). 2011 ACCF/AHA/SCAI Guideline for Percutaneous Coronary Intervention: A report of the American College of Cardiology Foundation/American Heart Association Task Force on Practice Guidelines and the Society for Cardiovascular Angiography and Interventions. Circulation.

[46] Budoff M.J., Dowe D., Jollis J.G., Gitter M., Sutherland J., Halamert E., Scherer M., Bellinger R., Martin A., Benton R. (2008). Diagnostic performance of 64-multidetector row coronary computed tomographic angiography for evaluation of coronary artery stenosis in individuals without known coronary artery disease: Results from the prospective multicenter ACCURACY (Assessment by Coronary Computed Tomographic Angiography of Individuals Undergoing Invasive Coronary Angiography) trial. J. Am. Coll. Cardiol..

[47] Meijboom W.B., Meijs M.F., Schuijf J.D., Cramer M.J., Mollet N.R., van Mieghem C.A., Nieman K., van Werkhoven J.M., Pundziute G., Weustink A.C. (2008). Diagnostic accuracy of 64-slice computed tomography coronary angiography: A prospective, multicenter, multivendor study. J. Am. Coll. Cardiol..

[48] Miller J.M., Rochitte C.E., Dewey M., Arbab-Zadeh A., Niinuma H., Gottlieb I., Paul N., Clouse M.E., Shapiro E.P., Hoe J. (2008). Diagnostic performance of coronary angiography by 64-row CT. N. Engl. J. Med..

[49] Tonino P.A., Fearon W.F., De Bruyne B., Oldroyd K.G., Leesar M.A., Ver Lee P.N., Maccarthy P.A., Van’t Veer M., Pijls N.H. (2010). Angiographic versus functional severity of coronary artery stenoses in the FAME study fractional flow reserve versus angiography in multivessel evaluation. J. Am. Coll. Cardiol..

[50] Min J.K., Shaw L.J., Devereux R.B., Okin P.M., Weinsaft J.W., Russo D.J., Lippolis N.J., Berman D.S., Callister T.Q. (2007). Prognostic value of multidetector coronary computed tomographic angiography for prediction of all-cause mortality. J. Am. Coll. Cardiol..

[51] Min J.K., Dunning A., Lin F.Y., Achenbach S., Al-Mallah M., Budoff M.J., Cademartiri F., Callister T.Q., Chang H.J., Cheng V. (2011). Age-and sex-related differences in all-cause mortality risk based on coronary computed tomography angiography findings results from the International Multicenter CONFIRM (Coronary CT Angiography Evaluation for Clinical Outcomes: An International Multicenter Registry) of 23,854 patients without known coronary artery disease. J. Am. Coll. Cardiol..

[52] Nakanishi R., Budoff M.J. (2016). Noninvasive FFR derived from coronary CT angiography in the management of coronary artery disease: Technology and clinical update. Vasc. Health Risk Manag..

[53] Sandevska E., Gjorcheva D.P., Vavlukis M., Sandevski A., Kafedziska I., Krstik-Damjanovska L., Majstorov V., Jovanovska-Perchinkova S., Guchev F., Kostova N. (2018). Myocardial Perfusion Abnormalities in Young and Premenopausal Women with Systemic Lupus Erythematosus, Detected with 99MTC MIBI Myocardial Perfusion Scintigraphy—Prevalence and Correlation with Proatherogenic Factors. Prilozi.

[54] Nayfeh M., Ahmed A.I., Saad J.M., Alahdab F., Al-Mallah M. (2023). The Role of Cardiac PET in Diagnosis and Prognosis of Ischemic Heart Disease: Optimal Modality Across Different Patient Populations. Curr. Atheroscler. Rep..

[55] Mavrogeni S.I., Markousis-Mavrogenis G., Karapanagiotou O., Toutouzas K., Argyriou P., Velitsista S., Kanoupakis G., Apostolou D., Hautemann D., Sfikakis P.P. (2019). Silent Myocardial Perfusion Abnormalities Detected by Stress Cardiovascular Magnetic Resonance in Antiphospholipid Syndrome: A Case-Control Study. J. Clin. Med..

[56] Burkard T., Trendelenburg M., Daikeler T., Hess C., Bremerich J., Haaf P., Buser P., Zellweger M.J. (2018). The heart in systemic lupus erythematosus—A comprehensive approach by cardiovascular magnetic resonance tomography. PLoS ONE.

[57] Mavrogeni S., Koutsogeorgopoulou L., Markousis-Mavrogenis G., Bounas A., Tektonidou M., Lliossis S.C., Daoussis D., Plastiras S., Karabela G., Stavropoulos E. (2018). Cardiovascular magnetic resonance detects silent heart disease missed by echocardiography in systemic lupus erythematosus. Lupus.

[58] Mavrogeni S., Bratis K., Markussis V., Spargias C., Papadopoulou E., Papamentzelopoulos S., Constadoulakis P., Matsoukas E., Kyrou L., Kolovou G. (2013). The diagnostic role of cardiac magnetic resonance imaging in detecting myocardial inflammation in systemic lupus erythematosus. Differentiation from viral myocarditis. Lupus.

[59] Mavrogeni S., Markousis-Mavrogenis G., Koutsogeorgopoulou L., Dimitroulas T., Bratis K., Kitas G.D., Sfikakis P., Tektonidou M., Karabela G., Stavropoulos E. (2017). Cardiovascular magnetic resonance imaging pattern at the time of diagnosis of treatment naïve patients with connective tissue diseases. Int. J. Cardiol..

[60] Zhang Y., Corona-Villalobos C.P., Kiani A.N., Eng J., Kamel I.R., Zimmerman S.L., Petri M. (2015). Myocardial T2 mapping by cardiovascular magnetic resonance reveals subclinical myocardial inflammation in patients with systemic lupus erythematosus. Int. J. Cardiovasc. Imaging.

[61] Leonard D., Svenungsson E., Sandling J.K., Berggren O., Jönsen A., Bengtsson C., Wang C., Jensen-Urstad K., Granstam S.O., Bengtsson A.A. (2013). Coronary heart disease in systemic lupus erythematosus is associated with interferon regulatory factor-8 gene variants. Circ. Cardiovasc. Genet..

[62] Leonard D., Svenungsson E., Dahlqvist J., Alexsson A., Ärlestig L., Taylor K.E., Sandling J.K., Bengtsson C., Frodlund M., Jönsen A. (2018). Novel gene variants associated with cardiovascular disease in systemic lupus erythematosus and rheumatoid arthritis. Ann. Rheum. Dis..

[63] Lundström E., Gustafsson J.T., Jönsen A., Leonard D., Zickert A., Elvin K., Sturfelt G., Nordmark G., Bengtsson A.A., Sundin U. (2013). HLA-DRB1*04/*13 alleles are associated with vascular disease and antiphospholipid antibodies in systemic lupus erythematosus. Ann. Rheum. Dis..

[64] Liu C., Zhou Y., Zhou Y., Tang X., Tang L., Wang J. (2023). Identification of crucial genes for predicting the risk of atherosclerosis with system lupus erythematosus based on comprehensive bioinformatics analysis and machine learning. Comput. Biol. Med..

[65] Gao N., Kong M., Li X., Wei D., Zhu X., Hong Z., Hong Z., Ni M., Wang Y., Dong A. (2022). Systemic Lupus Erythematosus and Cardiovascular Disease: A Mendelian Randomization Study. Front. Immunol..

[66] Kain J., Owen K.A., Marion M.C., Langefeld C.D., Grammer A.C., Lipsky P.E. (2022). Mendelian randomization and pathway analysis demonstrate shared genetic associations between lupus and coronary artery disease. Cell Rep. Med..

[67] Huang S., Huang F., Mei C., Tian F., Fan Y., Bao J. (2022). Systemic lupus erythematosus and the risk of cardiovascular diseases: A two-sample Mendelian randomization study. Front. Cardiovasc. Med..

[68] Svenungsson E., Gustafsson J., Leonard D., Sandling J., Gunnarsson I., Nordmark G., Jönsen A., Bengtsson A.A., Sturfelt G., Rantapää-Dahlqvist S. (2010). A STAT4 risk allele is associated with ischaemic cerebrovascular events and anti-phospholipid antibodies in systemic lupus erythematosus. Ann. Rheum. Dis..

[69] Øhlenschlaeger T., Garred P., Madsen H.O., Jacobsen S. (2004). Mannose-binding lectin variant alleles and the risk of arterial thrombosis in systemic lupus erythematosus. N. Engl. J. Med..

[70] Szalai A.J., Alarcón G.S., Calvo-Alén J., Toloza S.M., McCrory M.A., Edberg J.C., McGwin G., Bastian H.M., Fessler B.J., Vilá L.M. (2005). Systemic lupus erythematosus in a multiethnic US Cohort (LUMINA). XXX: Association between C-reactive protein (CRP) gene polymorphisms and vascular events. Rheumatology.

[71] Ding Y., Huang C., Zhao J., Wang Q., Tian X., Li M., Zeng X. (2023). The Impact of Antiphospholipid Antibodies on Future Atherosclerotic Cardiovascular Disease Risk in Systemic Lupus Erythematosus. Arthritis Rheumatol..

[72] Papazoglou N., Sfikakis P.P., Tektonidou M.G. (2024). Atherosclerotic Plaque Progression and Incident Cardiovascular Events in a 10-Year Prospective Study of Patients With Systemic Lupus Erythematosus: The Impact of Persistent Cardiovascular Risk Factor Target Attainment and Sustained DORIS Remission. Arthritis Rheumatol..

[73] Farina N., Abdulsalam R., McDonnell T., Pericleous C., D’Souza A., Ripoll V.M., Webster J., Isenberg D.A., Giles I., Rahman A. (2023). Antiphospholipid antibody positivity in early systemic lupus erythematosus is associated with subsequent vascular events. Rheumatology.

[74] Huang C., Ding Y., Chen Z., Wu L., Wei W., Zhao C., Yang M., Lin S., Wang Q., Tian X. (2025). Future atherosclerotic cardiovascular disease in systemic lupus erythematosus based on CSTAR (XXVIII): The effect of different antiphospholipid antibodies isotypes. BMC Med..

[75] Barinotti A., Radin M., Cecchi I., Foddai S.G., Arbrile M., Rubini E., Menegatti E., Roccatello D., Sciascia S. (2022). Assessing the cardiovascular risk in patients with systemic lupus erythematosus: QRISK and GAPSS scores head-to-head. Int. J. Cardiol..

[76] Nagy N., Bói B., Papp G., Fiák E., Gáspár-Kiss E., Perge B., Farmasi N., Tarr T. (2024). Antiphospholipid Antibodies Are Major Risk Factors for Non-Thrombotic Cardiac Complications in Systemic Lupus Erythematosus. Biomedicines.

[77] Patiño-Trives A.M., Pérez-Sánchez C., Pérez-Sánchez L., Luque-Tévar M., Ábalos-Aguilera M.C., Alcaide-Ruggiero L., Arias-de la Rosa I., Román-Rodríguez C., Seguí P., Espinosa M. (2021). Anti-dsDNA Antibodies Increase the Cardiovascular Risk in Systemic Lupus Erythematosus Promoting a Distinctive Immune and Vascular Activation. Arter. Thromb. Vasc. Biol..

[78] Logar D., Kveder T., Rozman B., Dobovisek J. (1990). Possible association between anti-Ro antibodies and myocarditis or cardiac conduction defects in adults with systemic lupus erythematosus. Ann. Rheum. Dis..

[79] Cao R., Lv H., Tang B., Lu Y. (2025). Anti-Ro/SSA antibodies in adult arrhythmias: Pathogenesis, clinical implications, and therapeutic strategies. Front. Immunol..

[80] Villuendas R., Martínez-Morillo M., Juncà G., Teniente-Serra A., Diez C., Heredia S., Riveros-Frutos A., Bayés-Genís A., Olivé A. (2021). Usefulness of cardiac screening in patients with systemic lupus erythematosus and anti-Ro/SSA antibodies. Lupus.

[81] Haque S., Skeoch S., Rakieh C., Edlin H., Ahmad Y., Ho P., Gorodkin R., Alexander M.Y., Bruce I.N. (2018). Progression of subclinical and clinical cardiovascular disease in a UK SLE cohort: The role of classic and SLE-related factors. Lupus Sci. Med..

[82] Ballocca F., D’Ascenzo F., Moretti C., Omedè P., Cerrato E., Barbero U., Abbate A., Bertero M.T., Zoccai G.B., Gaita F. (2015). Predictors of cardiovascular events in patients with systemic lupus erythematosus (SLE): A systematic review and meta-analysis. Eur. J. Prev. Cardiol..

[83] Katayama Y., Yanai R., Itaya T., Nagamine Y., Tanigawa K., Miyawaki Y. (2023). Risk factors for cardiovascular diseases in patients with systemic lupus erythematosus: An umbrella review. Clin. Rheumatol..

[84] Lu X., Wang Y., Zhang J., Pu D., Hu N., Luo J., An Q., He L. (2021). Patients with systemic lupus erythematosus face a high risk of cardiovascular disease: A systematic review and Meta-analysis. Int. Immunopharmacol..

[85] Sun E.Y., Alvarez C., Sheikh S.Z. (2019). Association of Lupus Nephritis With Coronary Artery Disease by ISN/RPS Classification: Results From a Large Real-world Lupus Population. ACR Open Rheumatol..

[86] Barbhaiya M., Feldman C.H., Guan H., Gómez-Puerta J.A., Fischer M.A., Solomon D.H., Everett B., Costenbader K.H. (2017). Race/Ethnicity and Cardiovascular Events Among Patients With Systemic Lupus Erythematosus. Arthritis Rheumatol..

[87] Garg S., Bartels C.M., Bao G., Helmick C.G., Drenkard C., Lim S.S. (2023). Timing and Predictors of Incident Cardiovascular Disease in Systemic Lupus Erythematosus: Risk Occurs Early and Highlights Racial Disparities. J. Rheumatol..

[88] Magder L.S., Petri M. (2012). Incidence of and risk factors for adverse cardiovascular events among patients with systemic lupus erythematosus. Am. J. Epidemiol..

[89] Tönnesmann E., Kandolf R., Lewalter T. (2013). Chloroquine cardiomyopathy—A review of the literature. Immunopharmacol. Immunotoxicol..

[90] Grimaldi L., Duchemin T., Hamon Y., Buchard A., Benichou J., Abenhaim L., Costedoat-Chalumeau N., Moride Y. (2024). Hydroxychloroquine and Cardiovascular Events in Patients With Systemic Lupus Erythematosus. JAMA Netw. Open.

[91] Choi M.Y., Li D., Feldman C.H., Yoshida K., Guan H., Kim S.C., Everett B.M., Costenbader K.H. (2021). Comparative risks of cardiovascular disease events among SLE patients receiving immunosuppressive medications. Rheumatology.

[92] Dedemadi A.G., Gkolfinopoulou C., Nikoleri D., Nikoloudaki M., Ruhanen H., Holopainen M., Käkelä R., Christopoulou G., Bournazos S., Constantoulakis P. (2025). Improvement of high-density lipoprotein atheroprotective properties in patients with systemic lupus erythematosus after belimumab treatment. Rheumatology.

[93] Sinicato N.A., da Silva Cardoso P.A., Appenzeller S. (2013). Risk factors in cardiovascular disease in systemic lupus erythematosus. Curr. Cardiol. Rev..

[94] Mok C.C., Birmingham D.J., Ho L.Y., Hebert L.A., Rovin B.H. (2013). High-sensitivity C-reactive protein, disease activity, and cardiovascular risk factors in systemic lupus erythematosus. Arthritis Care Res..

[95] Semalulu T., Tago A., Zhao K., Tselios K. (2023). Managing Cardiovascular Risk in Systemic Lupus Erythematosus: Considerations for the Clinician. ImmunoTargets Ther..

